# Foot-and-mouth disease virus downregulates vacuolar protein sorting 28 to promote viral replication

**DOI:** 10.1128/jvi.00181-23

**Published:** 2023-08-11

**Authors:** Xuefei Wang, Sahibzada Waheed Abdullah, Jin'en Wu, Jianli Tang, Yun Zhang, Hu Dong, Manyuan Bai, Sumin Wei, Shiqi Sun, Huichen Guo

**Affiliations:** 1 State Key Laboratory for Animal Disease Control and Prevention, National Foot-and-Mouth Disease Reference Laboratory, Lanzhou Veterinary Research Institute, Chinese Academy of Agricultural Sciences, Lanzhou, Gansu, China; 2 State Key Laboratory for Animal Disease Control and Prevention, College of Veterinary Medicine, Lanzhou University, Lanzhou, Gansu, China; University of Kentucky College of Medicine, Lexington, Kentucky, USA

**Keywords:** FMDV, Vps28, 2B, 3A, 3C^pro^, replication complex

## Abstract

Vacuolar protein sorting 28 (Vps28), a component of the ESCRT-I (endosomal sorting complex required for transport I), plays an important role in the pathogen life cycle. Here, we investigated the reciprocal regulation between Vps28 and the foot-and-mouth disease virus (FMDV). Overexpression of Vps28 decreased FMDV replication. On the contrary, the knockdown of Vps28 increased viral replication. Subsequently, the mechanistic study showed that Vps28 destabilized the replication complex (RC) by associating with 3A rather than 2C protein. In addition, Vps28 targeted FMDV VP0, VP1, and VP3 for degradation to inhibit viral replication. To counteract this, FMDV utilized tactics to restrict Vps28 to promote viral replication. FMDV degraded Vps28 mainly through the ubiquitin-proteasome pathway. Additional data demonstrated that 2B and 3A proteins recruited E3 ubiquitin ligase tripartite motif-containing protein 21 to degrade Vps28 at Lys58 and Lys25, respectively, and FMDV 3C^pro^ degraded Vps28 through autophagy and its protease activity. Meantime, the 3C^pro^-mediated Vps28 degradation principally alleviated the ability to inhibit viral propagation. Intriguingly, we also demonstrated that the N-terminal and C-terminal domains of Vps28 were responsible for the suppression of FMDV replication, which suggested the elaborated counteraction between FMDV and Vps28. Collectively, our results first investigate the role of ESCRTs in host defense against picornavirus and unveil underlying strategies utilized by FMDV to evade degradation machinery for triumphant propagation.

**IMPORTANCE:**

ESCRT machinery plays positive roles in virus entry, replication, and budding. However, little has been reported on its negative regulation effects during viral infection. Here, we uncovered the novel roles of ESCRT-I subunit Vps28 on FMDV replication. The data indicated that Vps28 destabilized the RC and impaired viral structural proteins VP0, VP1, and VP3 to inhibit viral replication. To counteract this, FMDV hijacked intracellular protein degradation pathways to downregulate Vps28 expression and thus promoted viral replication. Our findings provide insights into how ESCRT regulates pathogen life cycles and elucidate additional information regarding FMDV counteraction of host antiviral activity.

## INTRODUCTION

Foot-and-mouth disease virus (FMDV) belongs to the genus *Aphthovirus* within the viral family *Picornaviridae* and is the etiological agent of foot-and-mouth disease (FMD). FMD is a highly contagious disease in domestic and wild cloven-hoofed animals worldwide, including swine, sheep, goats, cattle, camelids, and deer (1, 2). It causes enormous economic losses in farming around the globe. FMDV contains a genome of approximately 8.5 kb, which encodes a single polyprotein that is subsequently processed into three structural proteins (VP0, VP1, and VP3) and eight nonstructural proteins (L^pro^, 2A, 2B, 2C, 3A, 3B, 3C^pro^, and 3D^pol^) by the leader protease (L^pro^) and 2A and 3C protease (3C^pro^). The capsid assembly is a vital step required for the production of the FMDV virion, which begins with the assembly of VP0, VP1, and VP3 to form the basic assembly subunit known as the protomer (5S). Five protomers subsequently assemble into pentamers (12S). Further assembly of 12 pentamers generates the 75S empty capsid. Finally, progeny RNA assembles into virions via encapsidation. VP0 undergoes a maturation cleavage to produce VP2 and VP4 (3), and the infectious virus is formed (146S).

The 2BC protein precursor is processed into 2B and 2C during FMDV infection, and it may play roles in immune evasion by contributing to persistent infections (4, 5). FMDV 2B protein comprises 154 amino acids and has a molecular weight of 17 kDa. It was previously determined that the 2B protein (also known as viroporin) triggers Nod-like receptor family pyrin domain containing 3 inflammasome activation and mediates the replication of FMDV (6), and antagonizes retinoic acid-inducible gene I-mediated antiviral effects via inhibition of its protein expression (7). Additionally, 2B protein interacts with laboratory of genetics and physiology 2, inhibiting its protein expression to exaggerate inflammatory response and promote viral replication (8). The 3A protein consists of 153 amino acids, which anchor the intracellular membrane of the host cell through its hydrophobic motif. 3A together with 2C protein is essential in replication complex (RC) formation, and 3A is involved in virulence and determining host range (9, 10). RC has been demonstrated to serve as the site for viral RNA synthesis (11), and it is important for viral replication. A previous study showed that FMDV used 3A protein to suppress detrimental factors. For instance, the interaction of FMDV 3A with DEAD-box helicase 56 reduced interferon regulatory factor 3 phosphorylation (12). Similarly, FMDV 3A interacts with annexin-A1 to inhibit interferon type I (IFN-I) production and create a favorable environment for FMDV replication (10). The 3C^pro^, which acts as a protease during FMDV replication, exhibited a decent inhibitory effect on host factors (13, 14). Although studies focusing on host–virus interaction assessed the impact of various FMDV proteins, 2B, 3A, and 3C^pro^ functions in the FMDV life cycle have yet to be fully elucidated.

The endocytic sorting complex required for transport (ESCRT) is an essential molecular machinery comprising more than 20 proteins. The complex formed by its components and their functional differences can be divided into five complexes, ESCRT-0, -I, -II, and -III, and vacuolar protein sorting 4 (Vps4) and some auxiliary proteins, such as ALG-2-interacting protein X (15). It plays critical roles in many physiological and metabolic processes, such as forming poly-vesicles, cytokinesis, plasma membrane repair, nuclear membrane reconstruction, and virus infection (16, 17). One of the essential functions of ESCRT is its participation in the life cycle of enveloped and nonenveloped viruses. Previous studies showed that the knockdown of ESCRT complexes hepatocyte growth factor–regulated tyrosine kinase substrate (Hrs), tumor susceptibility gene 101 (Tsg101), vacuolar protein sorting 25 (Vps25), and vacuolar protein sorting 24 (Vps24) inhibited cell entry of rotavirus (18). Similarly, ESCRT-0 component Hrs promoted the entry of Kaposi’s sarcoma-associated herpesvirus in human dermal microvascular endothelial cells (19). Recently, ESCRT machinery was found to help replicate the Classical swine fever virus (CSFV). Briefly, the ESCRT-I subunit Tsg101 interacted with nonstructural proteins NS4B and NS5B to form the CSFV RC (20). In addition, ESCRT takes part in the assembly and release of many viruses. The interaction between Tsg101 and VP40 of the Marburg virus helped assemble virus-like particles (VLPs) (21). Vps28, as a crucial element of ESCRT, also regulated various pathogens’ replication. Equine infectious anemia virus Gag protein association with Vps28 enhanced efficient release during infection (22). The knockdown of Vps28 increased hepatitis B virus release (23). Apart from the disparate function in releasing viruses, Vps28 also interacted with the influenza virus M1 protein to promote its replication (24).

The function of Vps28 on FMDV infection remains unknown. In the present study, we investigated the role of Vps28 during FMDV infection and determined its antiviral effect against FMDV. We also identified novel antagonistic mechanisms mediated by FMDV 2B, 3A, and 3C^pro^ proteins that inhibit Vps28-mediated antiviral activities.

## RESULTS

### Vps28 inhibits FMDV replication in PK-15 cells

**Fig 1:**
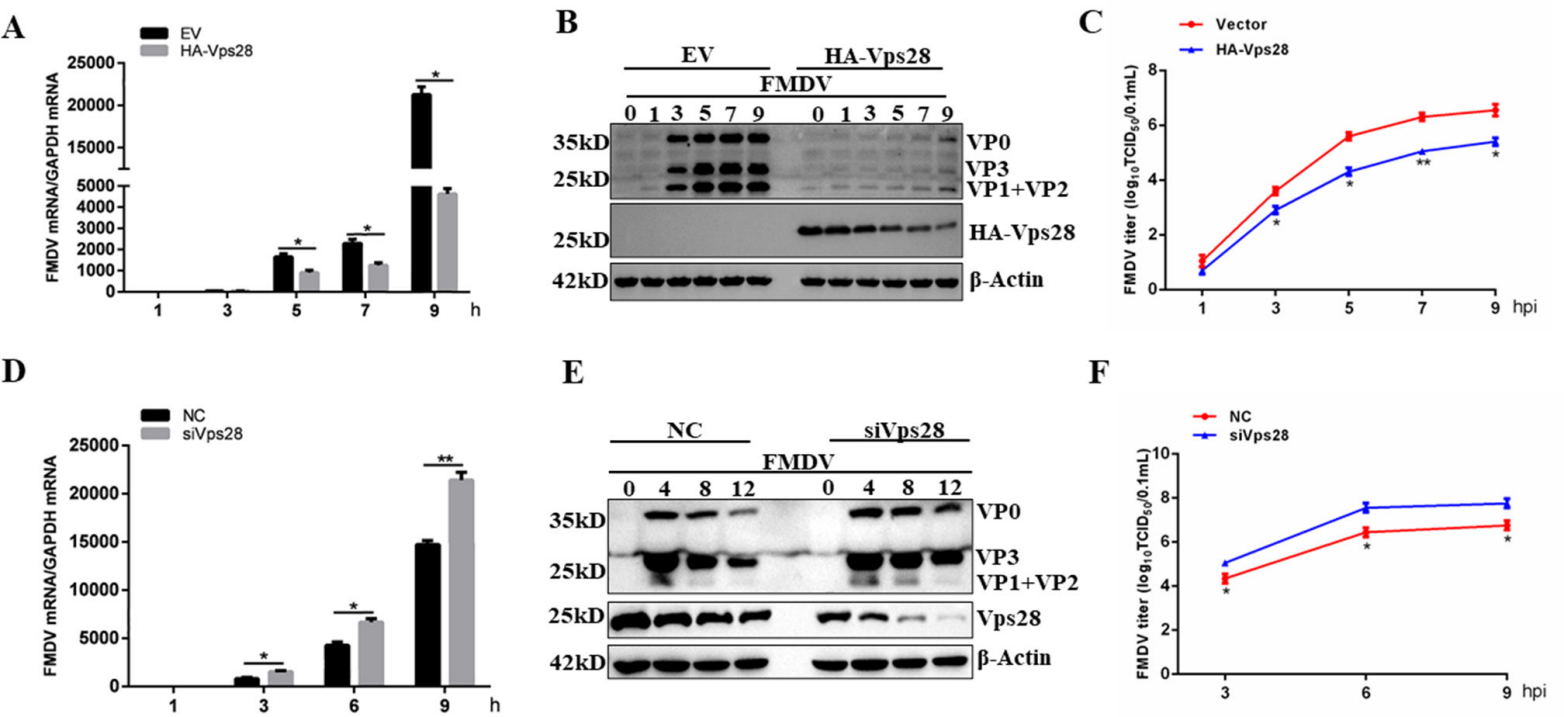
Vps28 inhibits FMDV replication in PK-15 cells. (A–C) PK-15 cells were transfected with plasmids encoding Vps28 for 24hours and then assigned to FMDV infection at a multiplicity of infection (MOI) of 1 for the indicated time points. The cell lysates were analyzed by western blot for FMDV protein level, and viral mRNA was measured using an RT-qPCR assay. The cell lysates and supernatants were collected, and viral yields were determined by a TCID_50_ assay. (D–F) siNC or siVps28 cells were infected with FMDV (MOI = 2) for the indicated time points. The cell samples were analyzed by western blot for viral protein level, and viral mRNA was measured using an RT-qPCR assay. The cell lysates and supernatants were collected, and viral yields were determined by a TCID_50_ assay. Data are means and SD of the results of three independent experiments. *P < 0.05; **P < 0.01.

A previous study showed that Vps28 played an important role in replicating FMDV in BHK-21 cells. Given that certain proteins may exert diverse functions in different cells, we utilized porcine kidney-15 (PK-15), the target cells of FMDV *in vivo*, to evaluate the effect of Vps28 in FMDV infection via silencing and overexpressing Vps28 expression. As shown in Fig. 1A-C, ectopic expression of Vps28 resulted in a concomitant decrease in FMDV replication. Consistent with the results, the knockdown of Vps28 enhanced viral replication (Fig. 1D-F). Finally, we examined the cytotoxicity of recombinant plasmids and small interfering RNA (siRNA) duplexes used in this study, and the results showed that the recombinant plasmids and siRNA duplexes were not toxic to cells (Fig. S1A and B). These results demonstrate that Vps28 exhibits an inhibitory effect on FMDV propagation.

### Vps28 destabilizes the replication complex of FMDV

**Fig 2:**
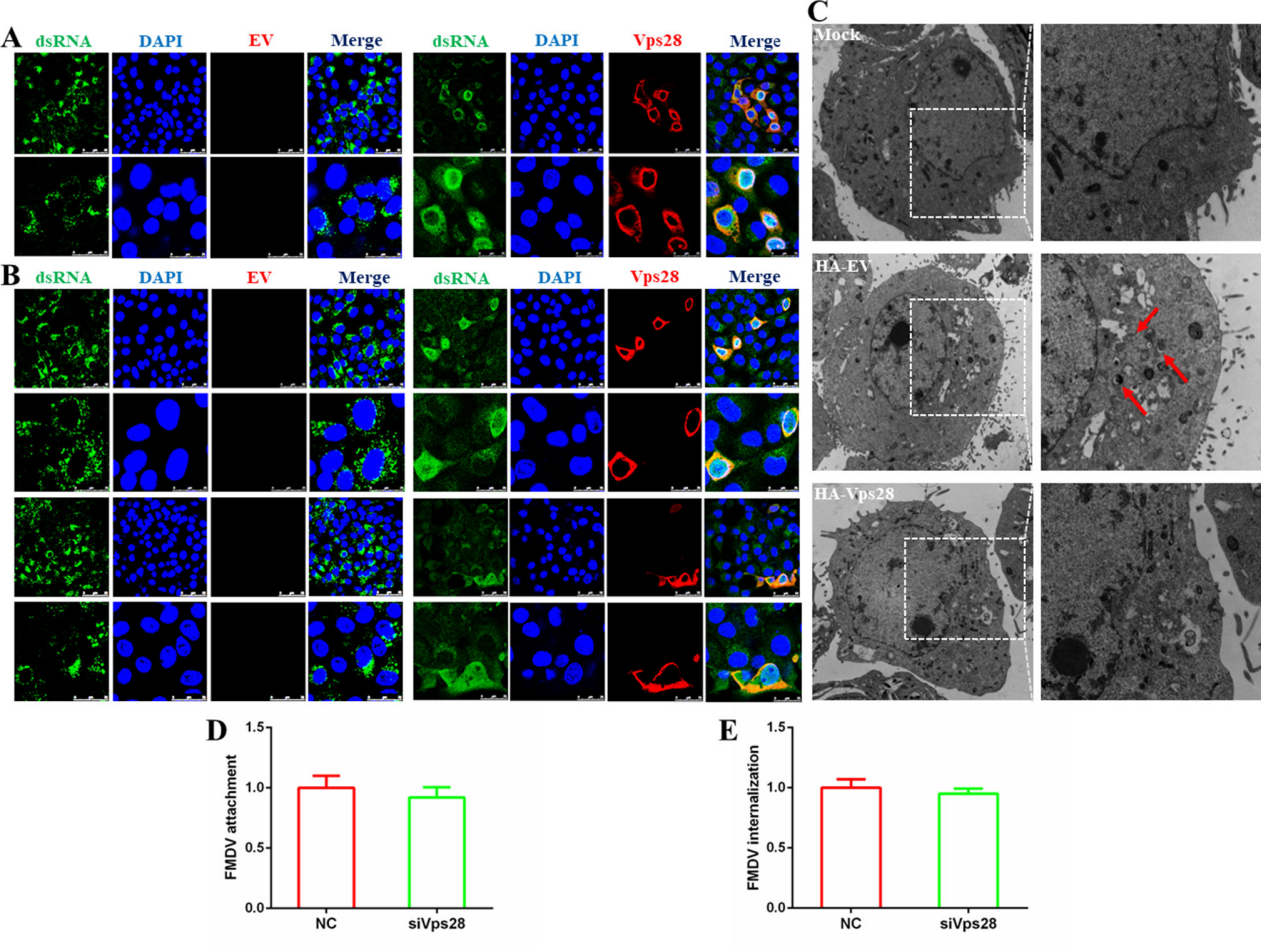
Vps28 destabilizes the replication complex of FMDV. (**A**) PK-15 cells transfected with HA (hemagglutinin)-EV or HA-Vps28 plasmids were infected with FMDV at an MOI of 1. At 3 hours post-infection (hpi), the cells were fixed and incubated with anti-dsRNA and anti-HA antibodies and then with secondary antibodies conjugated with FITC (green) and TRITC (red), respectively. Nuclei were counterstained with 4',6-diamidino-2-phenylindole (DAPI) (blue), and localization was determined using confocal microscopy. (**B**) PK-15 cells transfected with HA-EV or HA-Vps28 plasmids were infected with FMDV at an MOI of 1. At 3 and 5 hpi, the cells were fixed and incubated with anti-dsRNA and anti-HA antibodies and then with secondary antibodies conjugated with FITC (green) and TRITC (red), respectively. Nuclei were counterstained with DAPI (blue), and localization was determined using confocal microscopy. (**C**) PK-15 cells transfected with plasmids encoding HA-EV or HA-Vps28 were mock-infected and infected with FMDV at an MOI of 1, and images were obtained by transmission electron microscopy at 3hpi. Low-magnification images are shown in the left panel. High-magnification images are shown in the right panel. (**D**) siNC or siVps28 cells were infected with FMDV (MOI = 1) at 4°C for 1hour. The cell samples were collected and analyzed by RT-qPCR assay to determine the viral mRNA. (**E**) siNC or siVps28 cells were infected with FMDV (MOI = 1) at 37°C for 1hour. The cell samples were collected and analyzed by RT-qPCR assay to determine the viral mRNA.

Vps28 was recently reported to associate with the RC of CSFV (25). We hypothesized that Vps28 might regulate the FMDV RC as well. To test this, we first determined whether Vps28 co-localized with double-stranded RNA (dsRNA), a marker of the viral RC, to determine whether it is involved in the viral RC. The localization of Vps28 and dsRNA was assessed in FMDV-infected PK-15 cells. As shown in Fig. 2A, Vps28 strongly co-localized with dsRNA, demonstrating that Vps28 is related to the FMDV RC. We then evaluated the effects of Vps28 on the FMDV RC, and the results showed that Vps28 significantly decreased the dsRNA levels in 3hours (Fig. 2B, upper panel) and 5hours (Fig. 2B, lower panel) post-infection. Furthermore, TEM was performed to observe the RC generation in Vps28-transfected cells. Virus-induced modifications were observed in the perinuclear region (Fig. 2C, left panel, white boxes). The virus-induced membranous structures (vesicular clusters) were reduced in Vps28-transfected cells compared to EV-transfected cells. Double-membrane vesicles, which as the sites for RC anchoring and viral RNA synthesis (26), were also observed in EV-transfected cells (Fig. 2C, right panel, red arrows) but not in Vps28-transfected cells. Attachment and internalization are important events that happened before FMDV RNA replication (dsRNA) in the life cycle; when viral attachment and internalization are blocked, viral RNA replication decreases as well. Therefore, we evaluated the role of Vps28 in these two events to confirm its exclusive function before viral RNA replication. As shown in Fig. 2D and E, the knockdown of Vps28 had no influence on FMDV attachment and internalization, which confirmed that Vps28 did affect the RC generation. Collectively, these results indicated that Vps28 destabilizes the FMDV RC.

### Vps28 targets the 3A protein, a vital component of the FMDV replication complex

**Fig 3:**
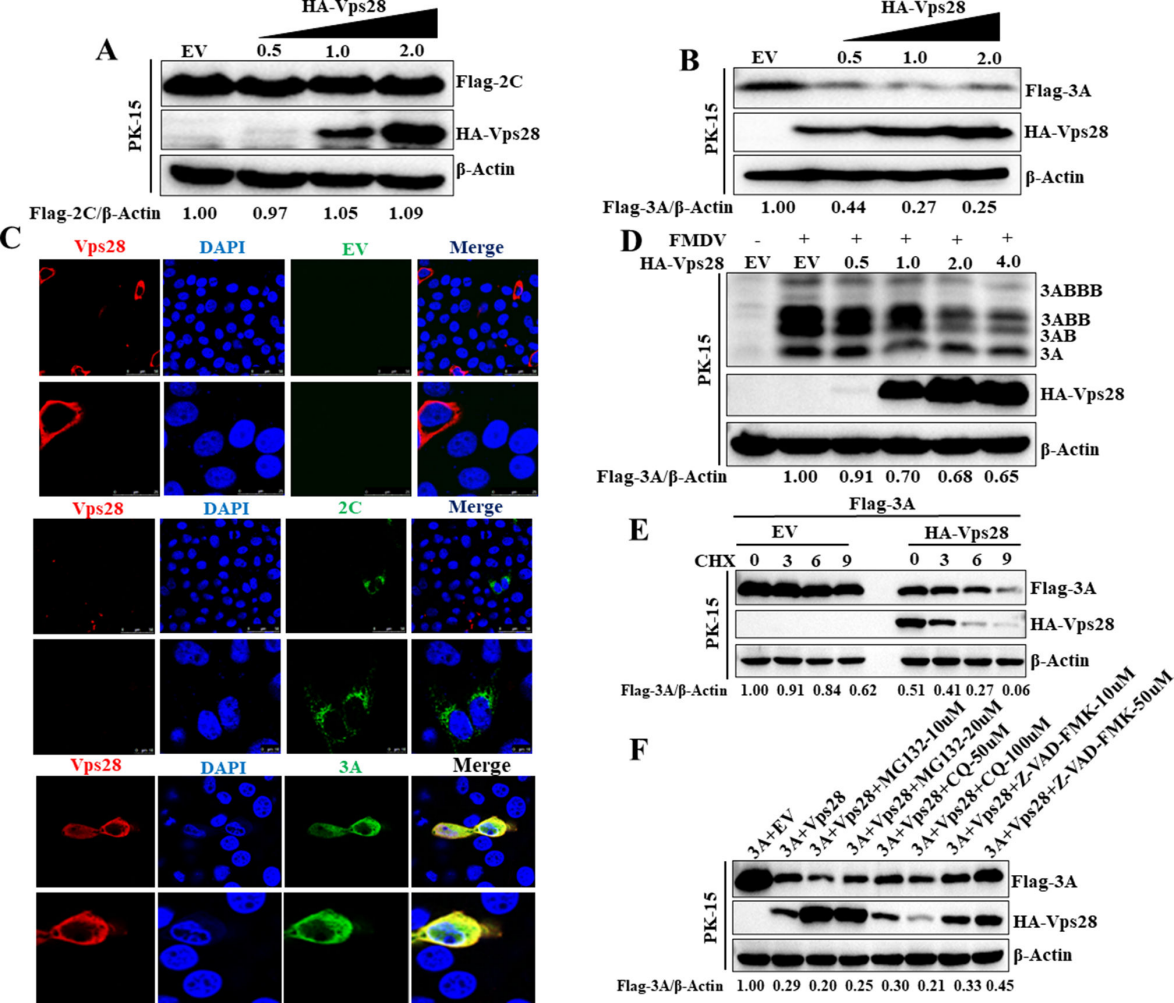
Vps28 targets the 3A protein, a vital component of the FMDV replication complex. (**A**) PK-15 cells were transfected with plasmids encoding HA-EV (2 µg) or HA-Vps28 (0.5, 1, and 2 µg) and Flag-2C (2 µg). Cell lysates were analyzed by western blot after 20 hours post-transfection (hpt). (**B**) PK-15 cells were transfected with plasmids encoding HA-EV (2 µg) or HA-Vps28 (0.5, 1, and 2 µg) and Flag-3A (2 µg). Cell lysates were analyzed by western blot after 20 hpt. (**C**) Plasmids encoding HA-Vps28 and Flag-EV or Flag-2C, or Flag-3A were co-transfected to PK-15 cells. At 18 hpt, the cells were fixed and incubated with anti-Flag and anti-HA antibodies and then with secondary antibodies conjugated with FITC (green) and TRITC (red), respectively. Nuclei were counterstained with DAPI (blue), and localization was determined using confocal microscopy. (**D**) PK-15 cells were transfected with plasmids encoding Vps28 for 24hours and then mock infected or infected with FMDV (MOI = 1) for 6hours. The cell lysates were analyzed by western blot. (**E**) PK-15 cells were co-transfected with HA-Vps28 or HA-EV, and Flag-3A plasmids, and the transfected cells were maintained in the culture medium in the presence of CHX (100 µg/mL), and the half-life of 3A protein was determined by western blot analysis in the presence or absence of Vps28. (**F**) The proteasome inhibitor MG132 (10 and 20 µM), the autophagy inhibitor CQ (50 and 100 µM), and the apoptosis inhibitor Z-VAD-FMK (10 and 50 µM) were added to PK-15 cells co-transfected with HA-Vps28 or HA-EV and Flag-3A after 6 hpt, and cell lysates were analyzed by western blot at 20 hpt.

Given that viral nonstructural proteins 2C and 3A are crucial components of the FMDV RC, relevant studies showed that Vps28 is related to the ubiquitination-proteasome system (27). Therefore, we analyzed whether Vps28 destabilizes the FMDV RC via targeting 2C or 3A. HA-Vps28 and Flag-2C or Flag-3A were co-transfected into PK-15 cells. We observed that 3A rather than 2C protein abundance was reduced by overexpression of Vps28 in a dose-dependent manner (Fig. 3A and B). Moreover, we found that Vps28 co-localized with 3A, but not 2C protein (Fig. 3C). Importantly, we also found that Vps28 downregulated FMDV 3A protein during the authentic infection (Fig. 3D). To further confirm the inhibition activity of Vps28 on 3A expression, PK-15 cells were co-transfected with HA-Vps28 and Flag-3A plasmids. The transfected cells were maintained in the culture medium in the presence of cycloheximide (CHX), and the half-life of 3A protein was determined by western blot analysis in the presence or absence of Vps28. As shown in Fig. 3E, Vps28 significantly accelerated the degradation of 3A in cells treated with CHX, indicating that Vps28 controls the half-life of 3A. The ubiquitin-proteasome system, the autophagy-lysosomal pathway, and apoptosis are three major intracellular protein degradation pathways in eukaryotic cells. To explore which pathway is responsible for the degradation of 3A by Vps28, the proteasome inhibitor carbobenzoxy-l-leucyl-l-leucyl-l-leucinal (MG132), the autophagy inhibitor chloroquine (CQ), and the apoptosis inhibitor carbobenzoxy-valyl-alanyl-aspartyl-(O-methyl)-fluoromethylketone (Z-VAD-FMK) were added to cells co-transfected with HA-Vps28 and Flag-3A, and 3A expression was analyzed by western blot. As shown in Fig. 3F, the incubation of Z-VAD-FMK restored 3A levels in Vps28 overexpressed cells, while MG132 and CQ treatment did not influence 3A restoration, demonstrating that Vps28 induced 3A degradation relied on apoptosis. The cytotoxicity of the inhibitors against PK-15 cells was determined using the CCK8 assay. All doses of the inhibitors used in the experiments showed no detectable cytotoxicity (Fig. S1C).

### Vps28 impairs FMDV structural proteins

**Fig 4:**
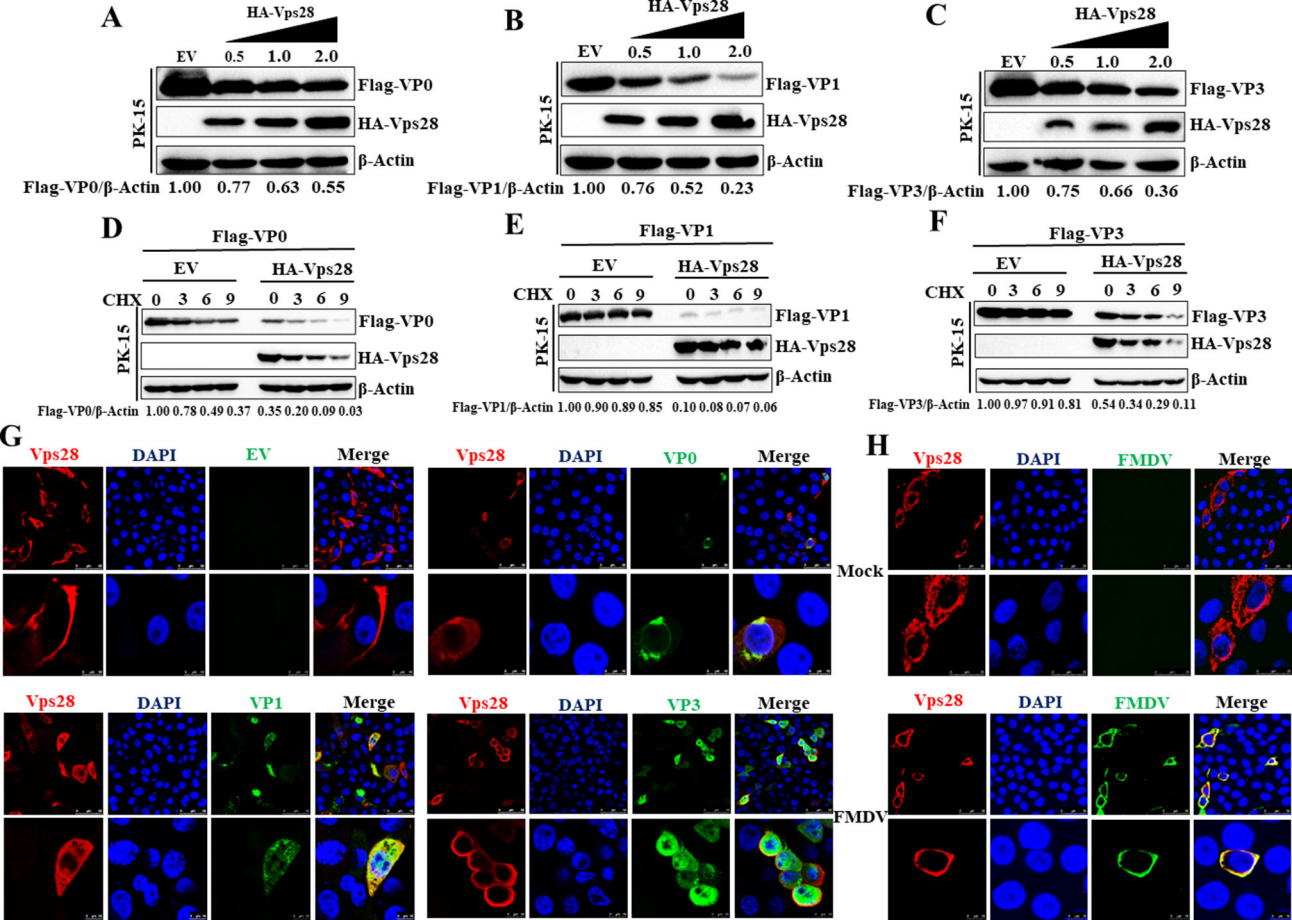
Vps28 impairs FMDV structural proteins. (A–C) PK-15 cells were co-transfected with plasmids encoding HA-EV (2 µg) or HA-Vps28 (0.5, 1, and 2 µg) and Flag-VP0 or Flag-VP1 or Flag-VP3 (2 µg). Cell lysates were analyzed by western blot after 20 hpt. (D–F) PK-15 cells were co-transfected with HA-Vps28 and Flag-VP0 or Flag-VP1, or Flag-VP3 plasmids. The transfected cells were maintained in the culture medium in the presence of CHX (100 µg/mL), and the half-life of VP0, VP1, and VP3 protein was determined by western blot analysis in the presence or absence of Vps28. (**G**) Plasmids encoding HA-Vps28 and Flag-VP0 or Flag-VP1 or Flag-VP3 were co-transfected to PK-15 cells. At 18 hpt, the cells were fixed and incubated with anti-Flag and anti-HA antibodies and then with secondary antibodies conjugated with FITC (green) and TRITC (red), respectively. Nuclei were counterstained with DAPI (blue), and localization was determined using confocal microscopy. (**H**) PK-15 cells transfected with plasmids encoding HA-Vps28 for 24hours were subject to FMDV infection. At 4 hpi, the cells were fixed and incubated with anti-FMDV and anti-HA antibodies and then with secondary antibodies conjugated with FITC (green) and TRITC (red), respectively. Nuclei were counterstained with DAPI (blue), and localization was determined using confocal microscopy.

We also assessed the effect of Vps28 on viral structural proteins. As mentioned above, HA-Vps28 and Flag-VP0 or Flag-VP1, or Flag-VP3 were co-transfected into PK-15 cells. Compared with HA-EV transfected cells, we found that VP0, VP1, and VP3 protein abundance was significantly reduced in HA-Vps28 transfected cells (Fig. 4A-C). To further confirm the inhibition activity of Vps28 on VP0, VP1, and VP3 expression, PK-15 cells were co-transfected with HA-Vps28 and Flag-VP0 or Flag-VP1 or Flag-VP3 plasmids. The half-life of VP0, VP1, and VP3 protein was investigated in CHX-treated HA-EV co-transfected cells and HA-Vps28 transfected cells. As shown in Fig. 4D-F, Vps28 significantly promoted the degradation of VP0, VP1, and VP3 in cells treated with CHX, indicating that Vps28 controls the half-life of VP0, VP1, and VP3. These results suggested that Vps28 impairs FMDV structural proteins. Furthermore, we assessed whether Vps28 co-localizes with VP0, VP1, and VP3 proteins. HA-Vps28 and Flag-VP0 or Flag-VP1, or Flag-VP3 were co-transfected into PK-15 cells, and the cells were fixed and subjected to confocal microscopy. The results showed that Vps28 co-localized with VP0, VP1, and VP3 proteins (Fig. 4G), besides, we also determined that Vps28 co-localized with FMDV structural proteins during authentic infection (Fig. 4H), which confirmed the regulation of Vps28 on FMDV structural proteins.

### FMDV degrades Vps28 mainly through the ubiquitin-proteasome pathway during viral infection

**Fig 5:**
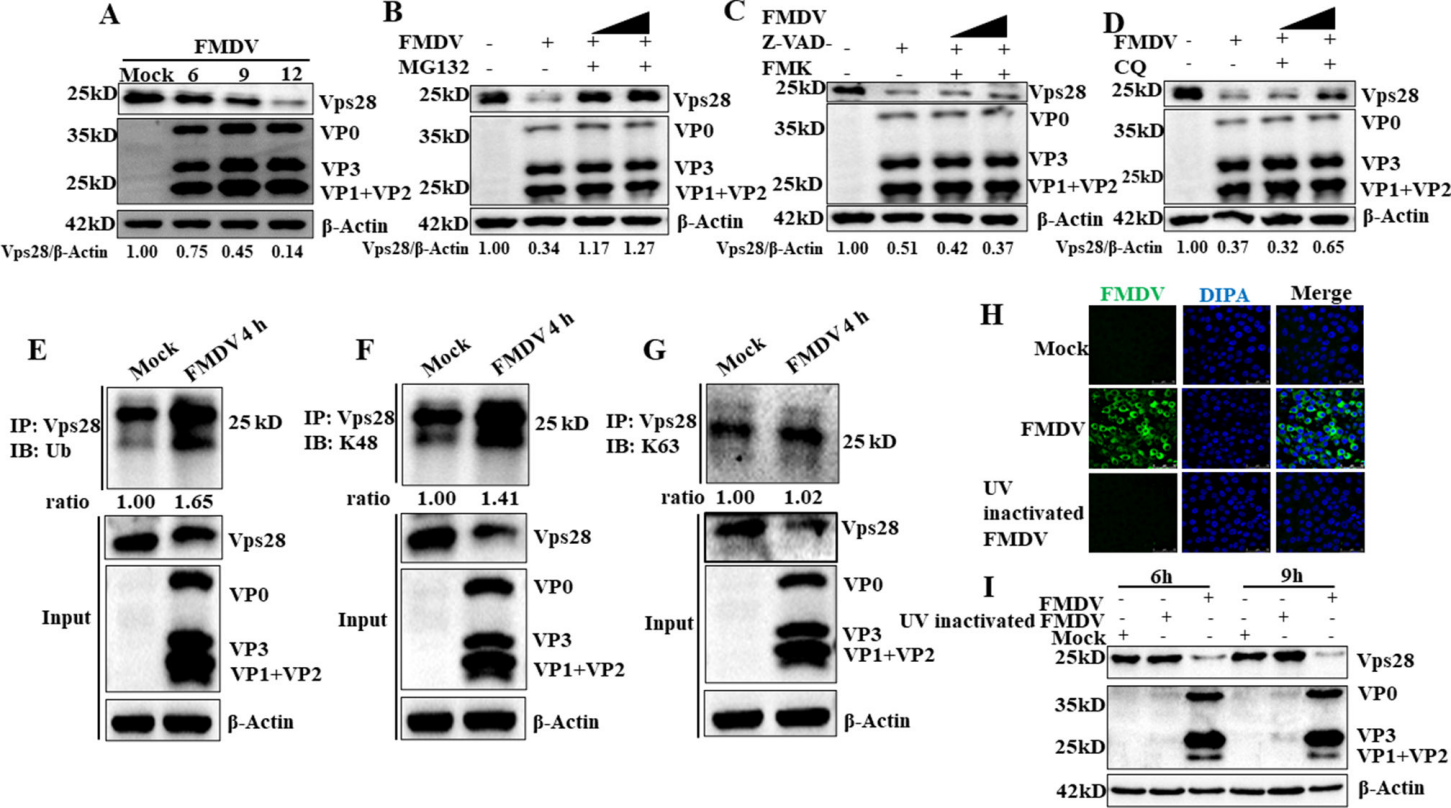
The ubiquitin-proteasome pathway plays a vital role in the degradation of Vps28 during FMDV infection. (**A**) PK-15 cells were infected with FMDV (MOI = 1). Cells were harvested 3hours intervals from 6 hpi, and dynamics of Vps28 were evaluated by western blot. (B–D) PK-15 cells were infected with FMDV (MOI = 1) and treated with MG132 (10 µM and 20 µM), CQ (50 µM and 100 µM), and Z-VAD-FMK (10 µM and 50 µM). At 9 hpi, cells were harvested, and the protein abundance of Vps28 was assessed by western blot. (E–G) PK-15 cells were infected with FMDV (MOI = 1), and cell lysates were collected at 4hpi. Cell lysates were incubated with anti-Vps28 antibody and protein G agarose. Whole-cell lysates and immunoprecipitates were analyzed by western blot using anti-Vps28, anti-ubiquitin, anti-K48, and anti-K63 antibodies. (**H**) PK-15 cells were infected with FMDV and UV-inactivated virus (MOI = 1). At 6 hpi, the cells were fixed and incubated with anti-FMDV serum and then with secondary antibodies conjugated with FITC (green). Nuclei were counterstained with DAPI (blue), and the fluorescence was determined using confocal microscopy. (**I**) PK-15 cells were infected with FMDV and UV-inactivated virus (MOI = 1), and cell lysates were collected at 6 hpi. Cell lysates were analyzed by western blot using anti-FMDV serum and anti-Vps28 antibodies.

To investigate the expression dynamics of Vps28 after FMDV infection, PK-15 cells were infected with FMDV strain China99 O at an MOI of 1, then the dynamics of Vps28 were analyzed by western blot at different time points (0, 6, 9, and 12hours) post-infection. The abundance of Vps28 protein was gradually decreased (Fig. 5A). To explore which pathway is responsible for the degradation of Vps28 during FMDV infection, the proteasome inhibitor MG132, the autophagy inhibitor CQ, and the apoptosis inhibitor Z-VAD-FMK were added to cells infected with FMDV, and Vps28 expression was analyzed by western blot. As shown in Fig. 5B-D, MG132 treatment completely restored the expression of Vps28 during FMDV infection. In addition, treatment with CQ mildly restored the expression of Vps28, while treatment with Z-VAD-FMK almost did not affect expression. These results suggested that the ubiquitin-proteasome pathway plays a major role in the degradation of Vps28 during FMDV infection. Based on the above results, we performed a co-immunoprecipitation (co-IP) assay to detect the ubiquitination level of Vps28 after FMDV infection. The results indicated that Vps28 was ubiquitinated after FMDV infection compared with uninfected cells (Fig. 5E). It is well known that the K48- and K63-linked polyubiquitin chains are the primary mediators of degradation. Therefore, we further analyzed Vps28 K48 and K63 ubiquitination levels in FMDV-infected cells. As expected, levels of Vps28 K48 ubiquitination were higher in cells infected with FMDV than in cells noninfected with FMDV (Fig. 5F), while Vps28 K63 ubiquitination kept the same in cells infected with FMDV and in cells noninfected with FMDV (Fig. 5G). These results showed that FMDV degrades Vps28 via the ubiquitin-proteasome pathway, and K48-linked ubiquitination is involved in this event. Finally, we analyzed whether the degradation of Vps28 depends on FMDV replication. To test this, we treated the FMDV with ultraviolet (UV), then PK-15 cells infected with normal FMDV or UV-treated FMDV for 6hours were subjected to confocal microscopy to analyze if we successfully obtained the inactivated virus. As shown in Fig. 5H, normal FMDV-infected PK-15 cells showed intense fluorescence, while UV-treated FMDV-infected PK-15 cells showed no fluorescence, suggesting we have successfully prepared the UV-inactivated virus that cannot replicate. Then PK-15 cells infected with FMDV and UV-inactivated FMDV were subjected to western blot. As shown in Fig. 5I, compared with mock-infected PK-15 cells, Vps28 expression significantly decreased in FMDV-infected PK-15 cells, while Vps28 reduction was not found in UV-inactivated FMDV-infected PK-15 cells, confirming that Vps28 is associated with FMDV replication.

### FMDV infection downregulates the Vps28 protein, dependent on FMDV 2B, 3A, and 3C^pro^ proteins

**Fig 6:**
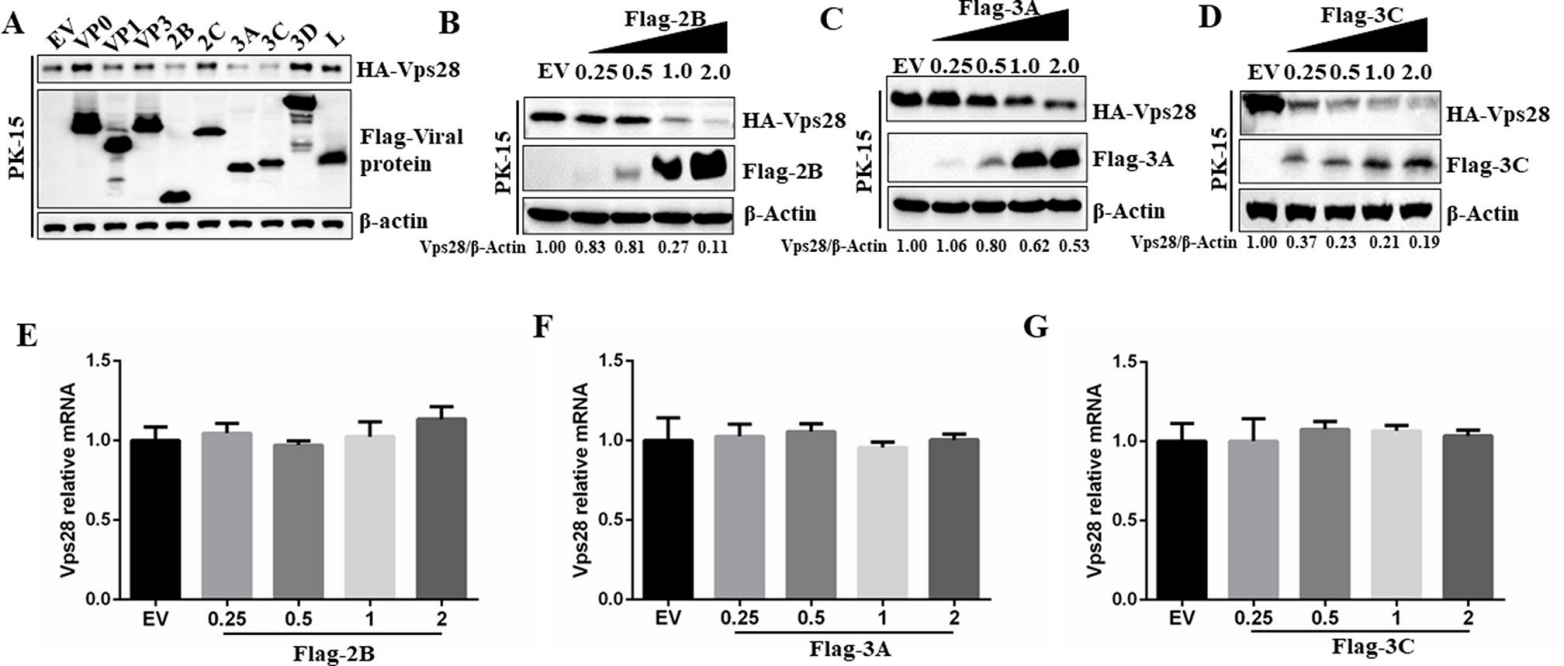
FMDV 2B, 3A, and 3C^pro^ proteins induced the degradation of Vps28. (**A**) PK-15 cells cultured in six-well plates were co-transfected with 2 µg plasmid expressing Flag-tagged viral proteins and 2 µg plasmid expressing HA-Vps28. At 24 hpt, the expression of HA-Vps28 proteins was assessed by western blot. (B–D) PK-15 cells cultured in six-well plates were co-transfected with Flag-EV (2 µg) or Flag-2B-, Flag-3A-, or Flag-3C^pro^-expressing plasmids (0.25, 0.5, 1, and 2 µg), and 2 µg plasmid expressing HA-Vps28 for 24 hours. Expression of the HA-Vps28 and viral proteins were detected by western blot. (E–G) PK-15 cells cultured in six-well plates were transfected with Flag-EV (2 µg) or Flag-2B-, Flag-3A-, or Flag-3C^pro^-expressing plasmids (0.25, 0.5, 1, and 2 µg), and Vps28 mRNA was determined by RT-qPCR assay at 24 hpt.

To further uncover the viral factors that influence Vps28 expression dynamics, PK-15 cells were co-transfected with HA-Vps28-expressing plasmid and plasmids expressing different Flag-tagged viral proteins. It was observed that the expression of 2B, 3A, and 3C^pro^ proteins significantly decreased Vps28 protein abundance (Fig. 6A). In addition, we co-transfected PK-15 cells with expression vectors encoding Vps28 and different doses of Flag-2B, Flag-3A, and Flag-3C and then assessed the protein expression of Vps28. 2B, 3A, and 3C^pro^ downregulated Vps28 expression at the protein level in a dose-dependent manner (Fig. 6B-D). We also transfected PK-15 cells with different doses of Flag-2B, Flag-3A, Flag-3C, and changes in Vps28 mRNA abundance were determined by RT-qPCR. As shown in Fig. 6E-G, 2B, 3A, and 3C^pro^ did not significantly affect the abundance of mRNA transcripts of Vps28. These results suggested that FMDV 2B, 3A, and 3C^pro^ mediated the downregulation of Vps28 expression not at the transcript level but through a protein degradation pathway.

### FMDV 2B, 3A protein degrades Vps28 via the ubiquitin-proteasome and apoptosis pathway

**Fig 7:**
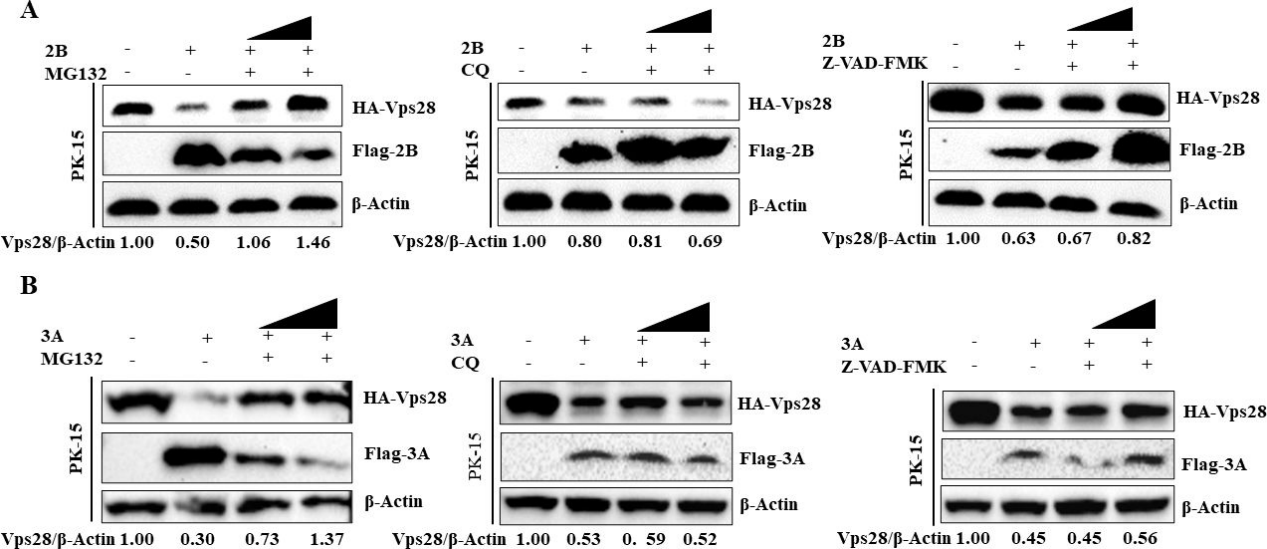
FMDV 2B, 3A protein degrades Vps28 via the ubiquitin-proteasome and apoptosis pathway. (**A**) PK-15 cells in six-well plates were co-transfected with HA-Vps28 (2 µg) and Flag-2B, or Flag-EV (2 µg). At 6hours post-transfection, cells were washed with 1× PBS, MG132 was added at 10–20 µM, CQ was added at 50–100 µM, and Z-VAD-FMK was added at 10–50 µM, and cells were incubated for an additional 18hours and collected in 1× SDS loading buffer. Samples were analyzed by western blot. (**B**) PK-15 cells in six-well plates were co-transfected with HA-Vps28 (2 µg) and Flag-3A, or Flag-EV (2 µg). At 6hours post-transfection, cells were washed with 1× PBS, MG132 was added at 10–20 µM, CQ was added at 50–100 µM, and Z-VAD-FMK was added at 10–50 µM, and cells were incubated for an additional 18hours and collected in 1× SDS loading buffer. Samples were analyzed by western blot.

Cells co-expressing 2B protein and Vps28 were treated with the ubiquitin-proteasome inhibitor MG132, the lysosomal inhibitor CQ, and the apoptosis inhibitor Z-VAD-FMK to identify the pathway of 2B protein downregulating Vps28. We found that treatment with MG132 and Z-VAD-FMK restored the expression of Vps28, while treatment with CQ did not affect the expression of Vps28 (Fig. 7A). Similarly, cells co-expressing 3A protein and Vps28 were treated with the ubiquitin-proteasome inhibitor MG132, the lysosomal inhibitor CQ, and the apoptosis inhibitor Z-VAD-FMK to assess the pathway through which 3A protein degrades Vps28. We found that treatment with MG132 and Z-VAD-FMK restored the expression of Vps28, while treatment with CQ did not affect the expression of Vps28 (Fig. 7B). These data indicated that FMDV 2B and 3A protein degraded Vps28 via the ubiquitin-proteasome and apoptosis pathways.

### Lys58 and Lys25 sites of Vps28 are responsible for its degradation by FMDV 2B and 3A protein, respectively

**Fig 8:**
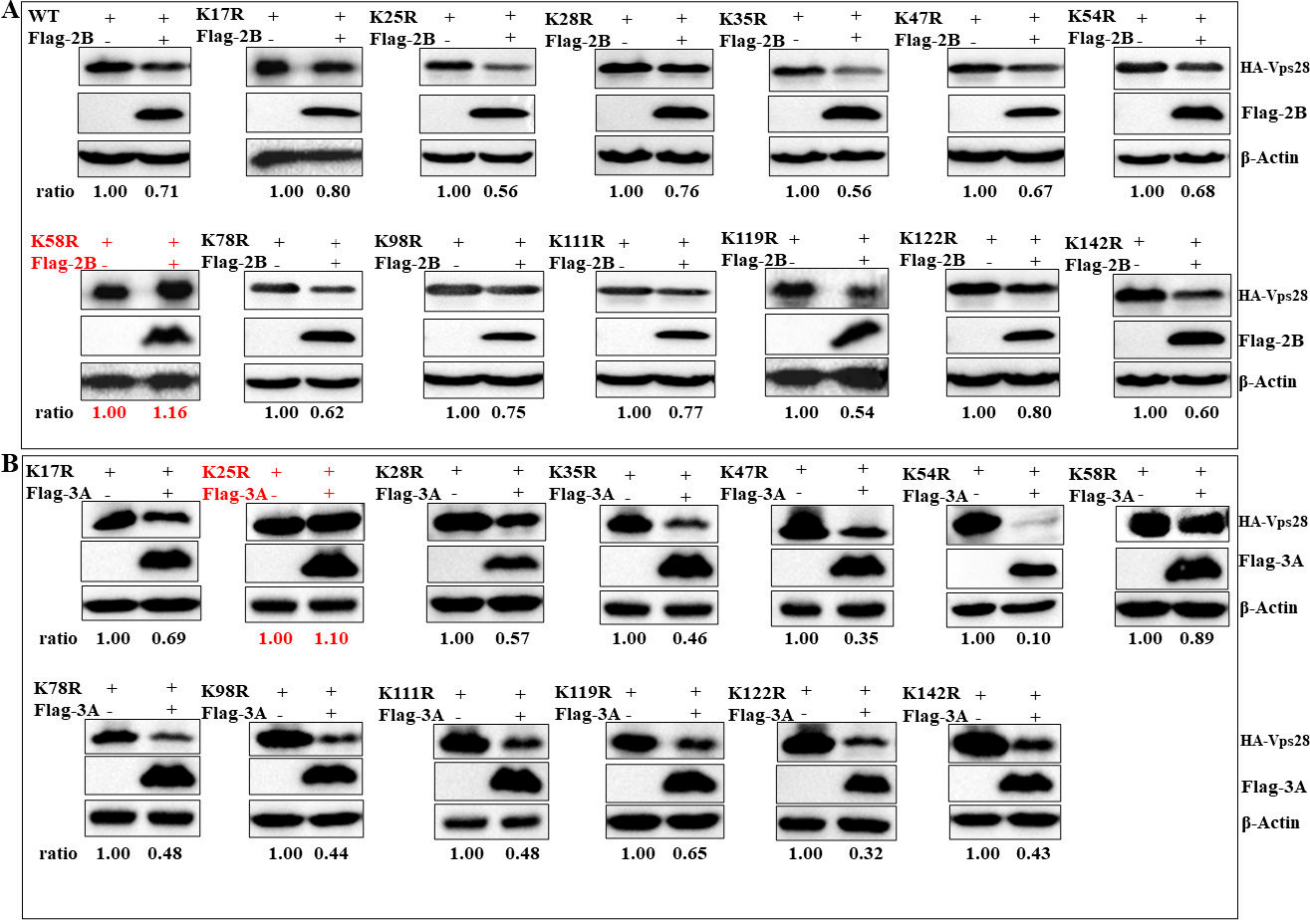
FMDV 2B and 3A protein target the Lys58 and Lys25 sites of Vps28 for its degradation, respectively. (**A**) PK-15 cells were co-transfected with HA-tagged wild-type (WT) Vps28 or Vps28 mutants (K17R, K25R, K28R, K35R, K47R, K54R, K58R, K78R, K98R, K111R, K119R, K122R, or K142R) and Flag-tagged FMDV 2B protein. After 24hours, cells were harvested to analyze Vps28 expression via western blot. (**B**) PK-15 cells were co-transfected with HA-tagged WT Vps28 or Vps28 mutants (K17R, K25R, K28R, K35R, K47R, K54R, K58R, K78R, K98R, K111R, K119R, K122R, or K142R) and Flag-tagged FMDV 3A protein. After 24hours, cells were harvested to analyze Vps28 expression via western blot.

To identify the potential sites targeted by FMDV 2B protein in Vps28, we co-transfected plasmids encoding Flag-2B protein and either wild-type HA-Vps28 or mutant HA-Vps28 (K17R, K25R, K28R, K35R, K47R, K54R, K58R, K78R, K98R, K111R, K119R, K122R, or K142R) in PK-15 cells. As shown in Fig. 8A, the K58R mutant completely abolished the degradation of Vps28 via 2B protein, suggesting that the Vps28 K58 site is targeted by FMDV 2B protein for degradation. To identify the possible sites targeted by FMDV 3A protein in Vps28, we co-transfected plasmids encoding Flag-3A protein and either wild-type HA-Vps28 or mutant HA-Vps28 (K17R, K25R, K28R, K35R, K47R, K54R, K58R, K78R, K98R, K111R, K119R, K122R, or K142R) in PK-15 cells. As shown in Fig. 8B, the K25R mutant completely abolished the degradation of Vps28 via 3A protein, suggesting that the Vps28 K25 site is targeted by FMDV 3A protein for degradation.

### FMDV 2B and 3A protein recruits E3 ubiquitin ligase TRIM21 to suppress Vps28 expression

**Fig 9:**
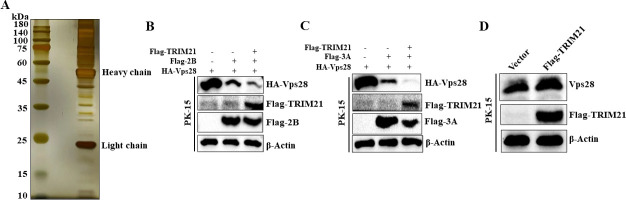
E3 ubiquitin ligase TRIM21 is recruited by FMDV 2B and 3A protein to suppress Vps28 expression. (**A**) Silver staining of the SDS-PAGE gel. (B and C) Plasmids encoding HA-Vps28, Flag-TRIM21, Flag-2B, or Flag-3A were co-transfected into PK-15 cells. Western blot was performed to detect protein expression at 24hours after co-transfection. (**D**) Plasmid encoding Flag-TRIM21 was transfected to PK-15 cells, and the expression of Vps28 was detected by western blot after 24hours of transfection.

Although we discovered that FMDV 2B and 3A proteins degrade Vps28 mainly through the ubiquitin-proteasome pathway, the E3 ubiquitin ligase involved in this event remains unclear. To clarify the E3 ligase, we performed a Co-IP/mass spectrometry assay and found that E3 ubiquitin ligases, including tripartite motif-containing protein 4 (TRIM4), tripartite motif-containing protein 21 (TRIM21), and ITCH, interact with Vps28. Silver staining exhibited a decent protein abundance (Fig. 9A). TRIM21 is a predominant E3 ubiquitin ligase recruited by enterovirus 71 (EV71) to mediate the proteasomal degradation of sterile alpha motif and histidine-aspartic acid domain-containing protein 1 (SAMHD1) (28). So we hypothesized that TRIM21 benefits the degradation of Vps28 mediated by 2B and 3A. To determine whether TRIM21 is recruited by 2B and 3A proteins to degrade Vps28, plasmids encoding HA-Vps28, Flag-TRIM21, and Flag-2B or Flag-3A were co-transfected into PK-15 cells. Western blot was performed to detect protein expression at 24hours after co-transfection. As shown in Fig. 9B and C, co-transfection of HA-Vps28, Flag-TRIM21, and Flag-2B or Flag-3A significantly decreased the expression of HA-Vps28 compared to HA-Vps28 and Flag-2B or Flag-3A co-transfected cells. We also transfected plasmids encoding Flag-TRIM21 to PK-15 cells to investigate if TRIM21 degrades Vps28 without 2B or 3A protein. It was observed that the expression level of Vps28 did not decrease (Fig. 9D). These results demonstrated that FMDV 2B and 3A proteins recruited E3 ubiquitin ligase TRIM21 to inhibit Vps28 expression.

### 3C^pro^ protease activity is required for Vps28 degradation

**Fig 10:**
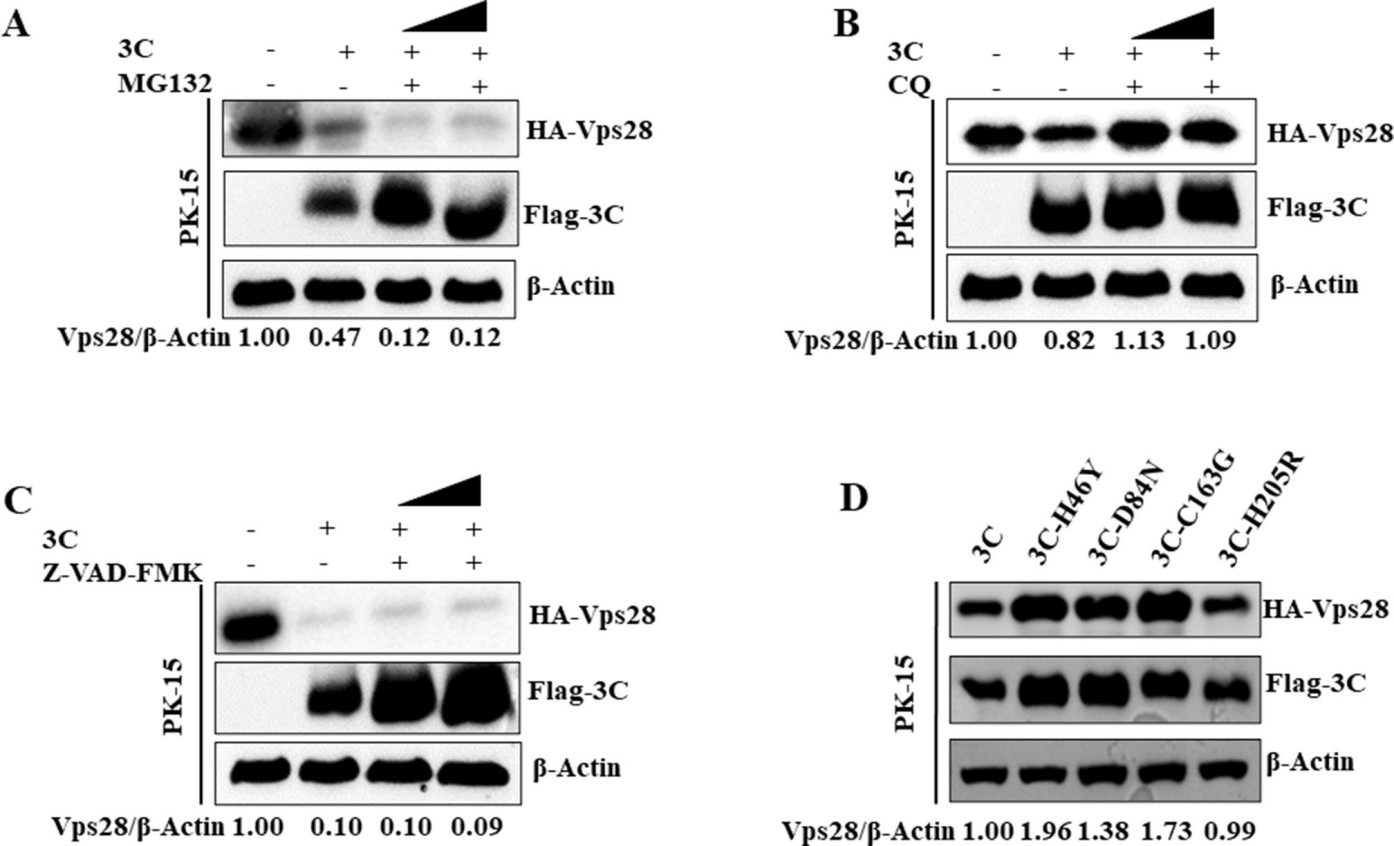
3C^pro^-induced degradation of Vps28 relies on its protease activity. (A–C) PK-15 cells in six-well plates were co-transfected with HA-Vps28 (2 µg) and Flag-3C or Flag-EV (2 µg). At 6hours post-transfection, cells were washed with 1× PBS, MG132 was added at 10–20 µM, CQ was added at 50–100 µM, and Z-VAD-FMK was added at 10–50 µM. Cells were incubated for 18hours, and samples were collected in 1× SDS loading buffer. Samples were analyzed by western blot. (**D**) PK-15 cells on six-well plates were co-transfected with HA-Vps28 (2 µg) and Flag-3C, H46Y, D84N, 163G, H205R, or FLAG-EV (2 µg). Samples were collected at 24hours post-transfection and analyzed by western blot.

The effects of MG132, CQ, or Z-VAD-FMK on 3C-induced degradation of Vps28 were assessed. As shown in Fig. 10A-C, treatment with CQ restored the expression of Vps28, while treatment with MG132 and Z-VAD-FMK showed no restoration of Vps28. To confirm whether the protease activity of 3C^pro^ was essential for the degradation of Vps28, we assessed 3C^pro^ mutants H46Y, D84N, and C163G, which lack protease activity. The mutant plasmid Flag-3C-H205R, which contains the enzymatic activity of 3C^pro^, was used as a control. PK-15 cells were co-transfected with HA-Vps28- and Flag-3C-expressing plasmid or Flag-3C^pro^ mutant plasmids. At 24 hpt, the expression level of Vps28 was determined by western blot. As shown in Fig. 10D, 3C^pro^ mutants lacking protease activity failed to relieve Vps28 abundance. These results demonstrated that the protease activity of 3C^pro^ was essential for the degradation of Vps28.

### FMDV 3C^pro^ protein principally alleviates the anti-FMDV effect of Vps28

**Fig 11:**
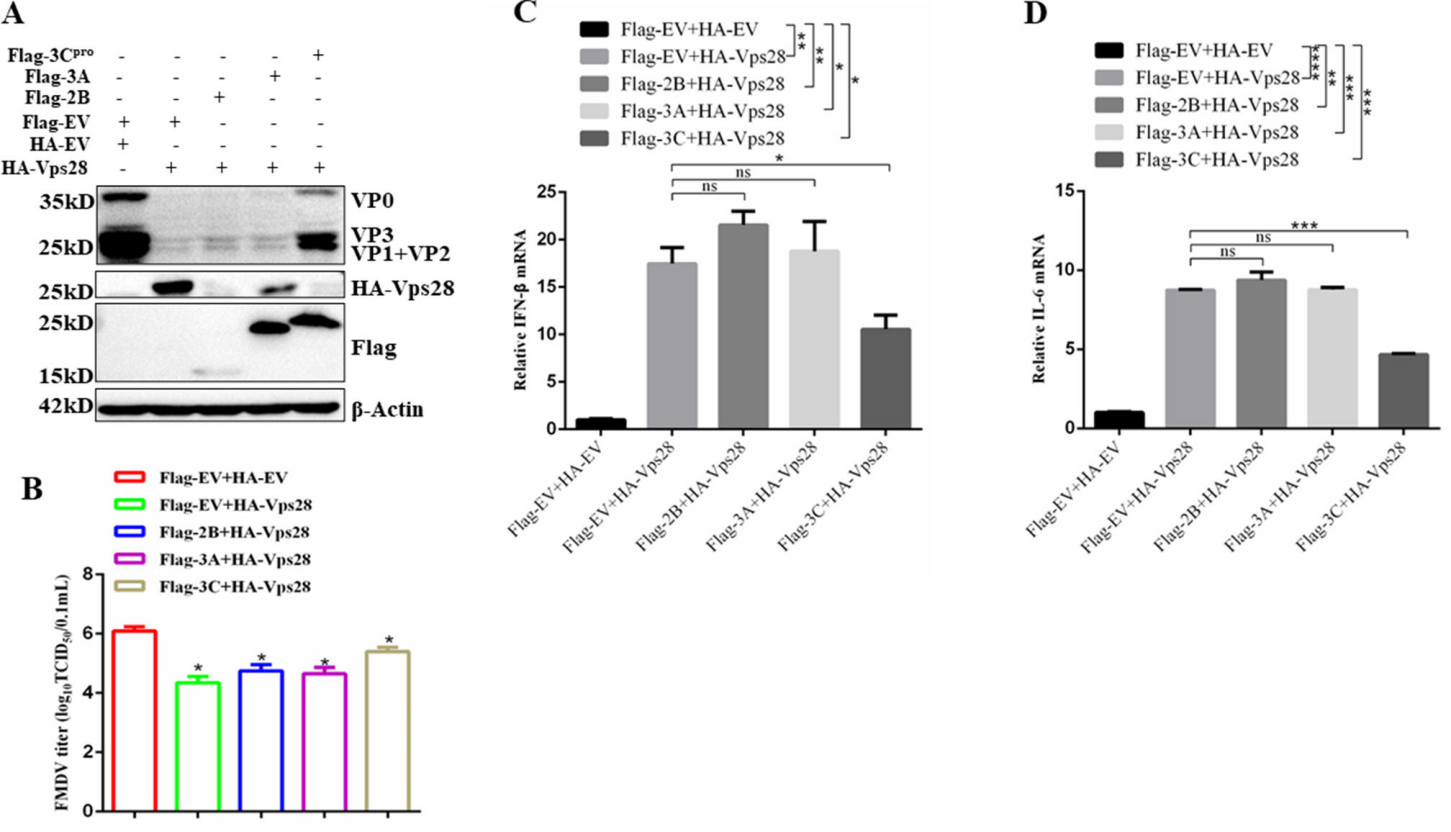
FMDV 3C^pro^ protein principally alleviates the anti-FMDV effect of Vps28. (A and B) PK-15 cells were co-transfected with Flag-EV and HA-EV or Flag-EV and HA-Vps28 or FLAG-2B and HA-Vps28 or Flag-3A and HA-Vps28, or Flag-3C and HA-Vps28 and then infected the cells with FMDV (MOI = 1). Cells were collected and then western blot and TCID_50_ assay were performed to detect protein expression and viral titer after FMDV infection. (C and D) PK-15 cells were transfected with Flag-EV and HA-EV or Flag-EV and HA-Vps28 or Flag-2B and HA-Vps28 or Flag-3A and HA-Vps28, or Flag-3C and HA-Vps28 and then infected the cells with FMDV (MOI = 1), 6 hours after FMDV infection, transcription levels of beta interferon (IFN-β) and IL-6 were detected by RT-qPCR. Data are means and SD of the results of three independent experiments. *P < 0.05; **P < 0.01; ***P < 0.001; ****P < 0.0001; ns, not significant.

Previous studies have illuminated that poly(I:C)-induced phosphorylation of TANK-binding kinase 1 (TBK1), p65, and IκBα was inhibited by knockdown of the ESCRT components, including Hrs, signal-transducing adaptor molecule 1 (STAM 1), signal-transducing adaptor molecule 2 (STAM 2), Tsg101, and Vps36. In addition, the knockdown of these components also attenuated the transcription of IFN-β1 and interferon-stimulated gene 56 (ISG56) genes induced by poly(I:C) (29). Vps28 is also a member of ESCRT, and Vps28 is correlated to FMDV replication. We hypothesized that Vps28 might also influence FMDV propagation via its antiviral activity, and the antiviral effects of Vps28 should be impaired by FMDV 2B, 3A, and 3C^pro^ protein. To test this hypothesis, we co-transfected PK-15 cells with Flag-EV and HA-EV or Flag-EV and HA-Vps28 or Flag-2B and HA-Vps28 or Flag-3A and HA-Vps28 or Flag-3C and HA-Vps28 and then infected the cells with FMDV (MOI = 1). Cells were collected, then western blot and TCID_50_ assay were performed to detect protein expression and viral titer 6hours after FMDV infection. As shown in Fig. 11A and B, the co-expression of 3C^pro^ protein significantly relieved the anti-FMDV activity of Vps28. To further explore the effects of 2B, 3A, and 3C^pro^-induced Vps28 downregulation on the innate immune responses mediated by FMDV, PK-15 cells were transfected with Flag-EV and HA-EV or Flag-EV and HA-Vps28 or Flag-2B and HA-Vps28 or Flag-3A and HA-Vps28 or Flag-3C and HA-Vps28 and then infected the cells with FMDV (MOI = 1). At 6hours after FMDV infection, transcription levels of IFN-β and IL-6 were detected. As shown in Fig. 11C and D, compared with Flag-EV and HA-Vps28 co-transfection, co-expression of Flag-3C and HA-Vps28 protein significantly alleviated the transcription levels of IFN-β and IL-6. These results indicated that FMDV 3C^pro^ protein principally alleviates the anti-FMDV effect of Vps28.

### FMDV degrades both N-terminal and C-terminal-Vps28

**Fig 12:**
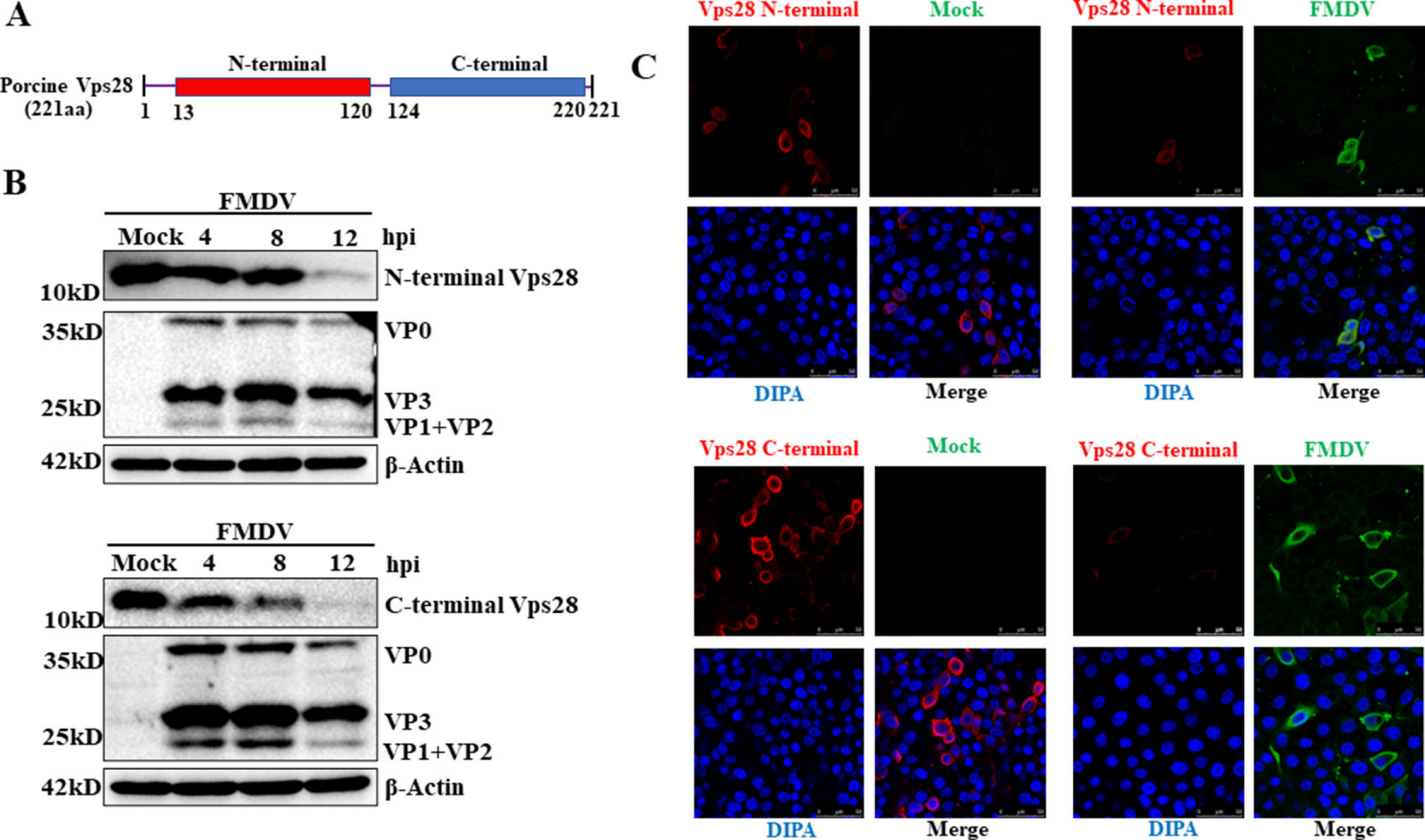
The concrete domain of Vps28 was degraded by FMDV. (**A**) Schematic representation of Vps28 mutant constructs. (**B**) PK-15 cells were transfected with HA-N-terminal-Vps28 and HA-C-terminal-Vps28 for 24hours, then infected with FMDV (MOI = 2) for the indicated time points, cells were collected, and cell lysates were analyzed by western blot. (**C**) Plasmids encoding HA-N-terminal-Vps28 and HA-C-terminal-Vps28 were transfected to PK-15 cells. At 24 hpt, cells were infected with FMDV (MOI = 1). Cells were fixed and incubated with anti-FMDV serum and anti-HA antibodies and then with secondary antibodies conjugated with FITC (green) and TRITC (red), respectively. Nuclei were counterstained with DAPI (blue), and localization was determined using confocal microscopy.

To further determine the certain domain degraded by FMDV, Vps28 was divided into N-terminal and C-terminal according to UniProt (Fig. 12A). We transfected PK-15 cells with HA-N-terminal-Vps28 and HA-C-terminal-Vps28, then infected with FMDV (MOI = 2). Cells were collected at the indicated time points and were analyzed by western blot. As shown in Fig. 12B, both N-terminal and C-terminal-Vps28 were degraded by FMDV. Furthermore, to confirm the degradation of N-terminal and C-terminal-Vps28 by FMDV, PK-15 cells were transfected with HA-N-terminal-Vps28 or HA-C-terminal-Vps28 and then infected with FMDV. The cells were fixed and subjected to an indirect immunofluorescence assay. As shown in Fig. 12C, the fluorescence intensity (red) was significantly decreased in FMDV-infected cells, compared with mock-infected cells.

### Vps28 depends on its crucial regions for antiviral activity during FMDV infection

**Fig 13:**
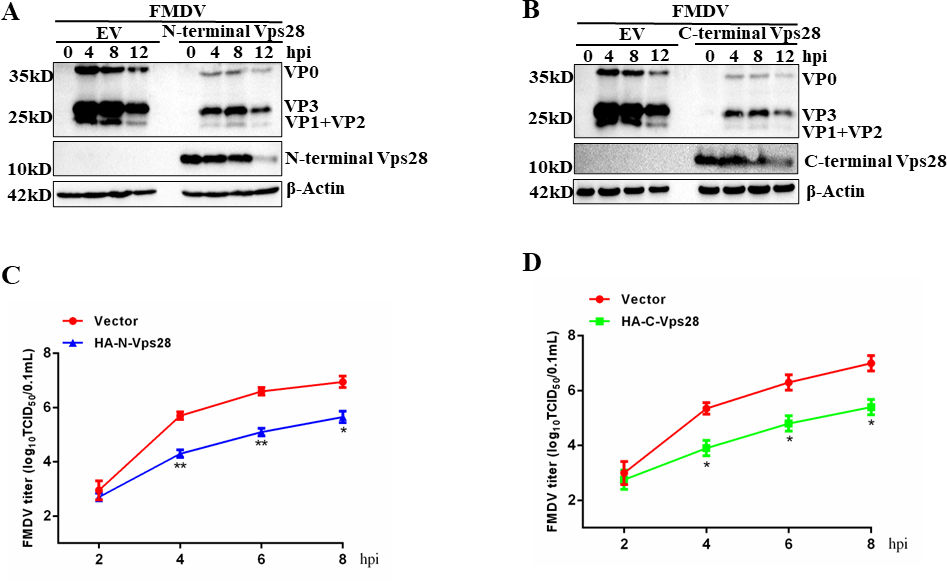
Functional domains of Vps28 suppress FMDV replication. (A–D) PK-15 cells were transfected with the empty vector (2 µg), HA-N-terminal-Vps28, or HA-C-terminal-Vps28 (2 µg) for 24hours and followed by infection with FMDV (2 MOI) for the indicated time points. The protein was detected by western blot, and viral titers were detected by TCID_50_ assay at the indicated time points. Data are means and SDs of the results of three independent experiments. *P < 0.05; **P < 0.01.

Next, the vital regions of Vps28 responsible for the suppression of FMDV replication were investigated. We transfected PK-15 cells with HA-N-terminal-Vps28 and HA-C-terminal-Vps28, then infected with FMDV (MOI = 2), cells were collected at the indicated time points, and western blot and TCID_50_ assay were performed to detect protein level and viral titers. As shown in Fig. 13A-D, both the N-terminal and C-terminal of Vps28 significantly inhibited protein expression levels and viral titers of FMDV.

## DISCUSSION

Viruses hijack cellular proteins to achieve their life cycle, including attachment, entry, replication, assembly, and release. Meanwhile, host cells utilize specific mechanisms to counter virus invasion. FMDV gets command of host cellular expression for its propagation, although various host factors also affect its life cycle. The underlying mechanisms by which FMDV proteins interact with host cell proteins have yet to be fully discovered.

**Fig 14:**
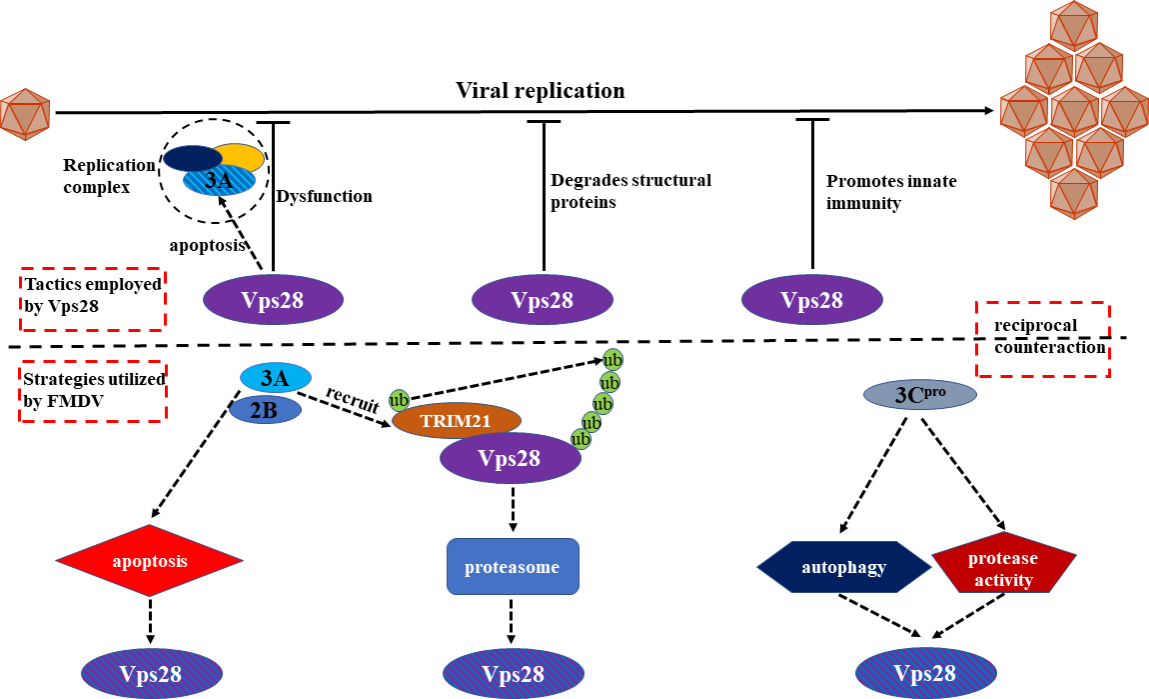
Schematic model depicting mutual counteraction between FMDV and Vps28. Vps28 suppresses FMDV replication by targeting 3A to destabilize the replication complex and degrading viral structural proteins VP0, VP1, and VP3. Meantime, innate immunity is promoted to inhibit the propagation of FMDV. To counteract this, FMDV 2B and 3A proteins hijack apoptosis and the ubiquitin-proteasome pathway in which E3 ubiquitin ligase TRIM21 is recruited to decrease Vps28 protein dynamics. Moreover, 3C^pro^ protease utilizes autophagy and its protease activity to degrade Vps28. Finally, the downgrading of Vps28 by FMDV promotes its replication.

ESCRTs classically functioned in the biogenesis of multivesicular bodies, the budding of HIV-1 and other viruses from the plasma membrane of infected cells, and the membrane abscission step in cytokinesis (30) were characterized to involve in diverse viral infection processes in the past few years. ESCRT complexes Hrs (ESCRT-0), Tsg101 (ESCRT-I), Vps25 (ESCRT-II), and Vps24 (ESCRT-III) enhanced cell entry of rotavirus (18). Alike, Hrs promoted the entry of Kaposi’s sarcoma-associated herpesvirus via macropinocytosis (19). Recently, ESCRT machinery was found to help replicate the CSFV (20, 25). In addition, ESCRTs also assemble the Japanese encephalitis virus (31, 32) and Marburg virus (21). Vps28, mainly related to the release of the equine infectious anemia virus (22), was soon discovered to facilitate the replication of the influenza virus (24). However, the mechanism of how Vps28 participates in the replication of picornavirus, especially FMDV, remains to be unveiled. Here, we investigated the role of Vps28 for FMDV replication and demonstrated that Vps28 suppresses FMDV replication by destabilizing the RC and viral structural proteins. Additionally, Vps28 also inhibits FMDV replication via promoting an innate immune response. To counteract this, FMDV 2B and 3A proteins hijack apoptosis and the ubiquitin-proteasome pathway in which E3 ubiquitin ligase TRIM21 is recruited to decrease Vps28 protein dynamics. Moreover, 3C^pro^ protease utilizes autophagy and its protease activity to degrade Vps28. Finally, the downgrading of Vps28 by FMDV promotes its replication (Fig. 14).

Although it is generally reckoned that viruses hijack cellular components to promote their propagation in host cells, it is interesting to note that cellular components are sometimes associated with antiviral activity. Our result demonstrated that overexpression of Vps28 significantly decreased viral yields, and knockdown of Vps28 enhanced FMDV replication in PK-15 cells. Depletion of Vps28 possessed limited function compared to ectopic expression, which may be because the existence of Vps28 isoforms could mask the effect of silencing the expression of this protein.

The stability of viral proteins is essential for virus replication. A previous study in our laboratory showed that heat shock protein 60 (Hsp60) interacted with FMDV nonstructural proteins 2C and 3A to maintain its stability and enhance the replication of FMDV (9). *N*-acetyltransferase 8 promoted viral replication by increasing the stability of EV71 nonstructural proteins 2B, 3AB, and 3C (33). E2 ubiquitin-conjugating enzyme UBE2L6 promotes Senecavirus A proliferation by stabilizing the viral RNA polymerase (34). Consistently, the destabilization of viral protein inhibits the replication of the virus. The E3 ubiquitin ligase TBK1 mediated the degradation of FMDV VP3 proteins, thus inhibiting the replication of FMDV (35). Cellular dnaJ heat shock protein family (Hsp40) member A3 (DNAJA3) inhibits FMDV replication by destabilizing VP1 (2). Selective autophagy receptor SQSTM1/p62 inhibits Seneca Valley virus replication by targeting viral VP1 and VP3 for degradation (36). Intriguingly, one of the main functions of ESCRTs and subunits is to select and degrade cargo (30). Based on the above studies, we hypothesized that Vps28 regulates viral protein function to inhibit the replication of FMDV. Our result showed that Vps28 co-localized with 3A protein and regulated the stability of viral nonstructural protein 3A. Most importantly, the 3A protein is a key component of FMDV RC (9, 37, 38), and the RC dysfunction causes the suppression of the viral replication. Therefore, RC dysfunction is one mechanism for inhibiting FMDV propagation by Vps28. Interestingly, Vps28 also limited the expression of FMDV structural proteins VP0, VP1, and VP3.

The ubiquitin-proteasome system, the autophagy-lysosomal pathway, and apoptosis are three major intracellular protein degradation pathways in eukaryotic cells. To determine which pathway is responsible for the degradation of Vps28 during FMDV infection, the proteasome inhibitor MG132, the autophagy inhibitor CQ, and the apoptosis inhibitor Z-VAD-FMK were added to cells infected with FMDV. We found that MG132 treatment completely restored the expression of Vps28 during FMDV infection. Treatment with CQ mildly restored the expression of Vps28. In contrast, treatment with Z-VAD-FMK almost did not affect expression. These results suggested that the ubiquitin-proteasome pathway plays a major role in the degradation of Vps28 during FMDV infection. On this basis, a Co-IP assay was performed to detect the ubiquitination level of Vps28 after FMDV infection, and the results indicated that Vps28 was ubiquitinated after FMDV infection compared with uninfected cells. Moreover, we confirmed that FMDV contributes to Vps28 degradation in a K48-linked ubiquitin-proteasome-mediated manner.

Further investigation showed that Vps28 protein expression was reduced by FMDV 2B, 3A, and 3C^pro^. Additionally, we transfected 2B, 3A, and 3C^pro^ in a concentration-increasing manner, which resulted in a consistent decrease in Vps28 protein levels. In the current study, we also investigated the involvement of the ubiquitin-proteasome, the autophagy-lysosomal, and apoptosis pathway in FMDV 2B-, 3A-, and 3C^pro^ -dependent degradation of Vps28, which showed that FMDV 2B and 3A mainly use the ubiquitin-proteasome pathway to degrade Vps28. Site analysis indicated that Lys58 and Lys25 of Vps28 are the critical amino acid sites responsible for Vps28 degradation by 2B and 3A, respectively. Although we discovered that FMDV 2B and 3A proteins degrade Vps28 mainly through the ubiquitin-proteasome pathway, the E3 ubiquitin ligase involved in this event remains unclear. To clarify the E3 ligase, we performed a Co-IP/mass spectrometry assay and found that E3 ubiquitin ligases, including TRIM4, TRIM21, and ITCH, interact with Vps28. TRIM21 is a predominant E3 ubiquitin ligase recruited by EV71 to mediate the proteasomal degradation of SAMHD1. We hypothesized that TRIM21 benefits the degradation of Vps28 mediated by 2B and 3A. Our results showed that 2B and 3A recruit E3 ubiquitin ligase TRIM21 to degrade Vps28.

In contrast, 3C^pro^ utilized the autophagy-lysosomal pathway to degrade the Vps28 protein. The different protein degradation pathways used by 2B, 3A, and 3C^pro^ suggest that host proteins may be degraded due to the unique behavior of FMDV proteins. 3C^pro^ was reported to inhibit host protein expression via its catalytical activity during FMDV infection (39). The present study indicates that FMDV 3C^pro^ results in Vps28 degradation, partly due to the enzymatic activity of 3C^pro^. Vps28 degradation was not observed in catalytically inactive 3C^pro^ mutants such as H46Y, D84N, and C163G, although the constitutively catalytically active mutant H205R showed degradation of Vps28 similar to wild-type 3C^pro^ levels. Interestingly, among these FMDV proteins, the degradation of Vps28 by 3C^pro^ principally alleviated the anti-FMDV effect of Vps28, which confirmed that 3C^pro^ is mainly responsible for reducing host proteins exerting an antiviral effect.

Vps28 comprises at least two domains, an N-terminal region responsible for oligomerization and a C-terminal domain that folds autonomously into a four-helical bundle structure. The previous study has shown that the C-terminal domain of Vps28 rescued an equine infectious anemia virus Gag late domain mutant, resulting in a clear release of Gag into the supernatant (40). We also divided swine Vps28 into the N-terminal domain and C-terminal domain according to UniProt and found that both the N-terminal domain and C-terminal domain significantly inhibited the propagation of FMDV. Due to the elaborate interaction between FMDV and Vps28, FMDV decreased both the N-terminal domain and C-terminal of Vps28.

In summary, we initiatively assessed the function of ESCRT-I subunit Vps28, in the replication of picornavirus and showed that Vps28 inhibited FMDV replication via promoting innate immune response and destabilizing the viral RC and structural proteins. Although Vps28 exhibits antiviral activity, FMDV has also developed an elaborate strategy to antagonize the effect of Vps28, highlighting the complex interactions between FMDV and host Vps28.

## MATERIALS AND METHODS

### Cells, viruses, and infection

PK-15 cells (porcine kidney; ATCC CCL-33) were maintained in Dulbecco’s modified Eagle’s medium (DMEM) (Gibco, California, USA) supplemented with 10% fetal bovine serum (FBS) (Gibco), penicillin (100 U/mL), and streptomycin (100 mg/mL) (Gibco) at 37°C under 5% CO_2_. FMDV serotype O strain O/China/99 (GenBank accession no. AF506822.2) was maintained by the OIE/National Foot-and-Mouth Disease Reference Laboratory (Lanzhou, China). FMDV was propagated in BHK-21 cells, and the viral titers were determined with a 50% tissue culture infective dose (TCID_50_) assay in BHK-21 cells.

### Antibodies and reagents

Rabbit anti-Vps28 monoclonal antibody was purchased from Abcam (Cambridge, MA, USA). Mouse anti-Flag monoclonal antibody and rabbit anti-HA polyclonal antibody were purchased from Proteintech (Wuhan, China). Mouse anti-HA monoclonal antibody was purchased from Thermo Fisher Scientific (Waltham, MA, USA). Mouse anti-actin monoclonal antibody was purchased from CWBIO (Beijing, China). Anti-FMDV structural protein polyclonal pig antiserum was prepared by our laboratory by immunizing pigs with FMDV (O/BY/CHA/2010; GenBank accession no. JN998085.1) VLP. Anti-FMDV nonstructural protein 3A polyclonal rabbit antiserum was prepared by our laboratory by immunizing rabbits with FMDV (O/BY/CHA/2010; GenBank accession no. JN998085.1) nonstructural protein 3A. The secondary antibodies conjugated with horseradish peroxidase (HRP), fluorescein isothiocyanate (FITC), and tetramethyl rhodamine isocyanate (TRITC) were purchased from Sigma-Aldrich (St. Louis, MO, USA). Lipofectamine RNAiMAX and Lipofectamine 2000 were purchased from Invitrogen (California, USA). MG132, CQ, Z-VAD-FMK, and CHX were purchased from MedChemExpress (Monmouth Junction, New Jersey, USA). All drugs in this study were dissolved in 0.1% dimethyl sulfoxide (DMSO).

### Plasmid constructs

Mammalian expression plasmids for the FMDV structural proteins VP0, VP1, and VP3 and nonstructural proteins 2B, 2C, 3A, 3C^pro^, 3D^pol^, L^pro^,3C-H46Y, 3C-D84N, 3C-163G, and 3C-H205R were previously synthesized by our laboratory (41, 42). HA-tagged Vps28, HA-tagged N-terminal Vps28 and HA-tagged C-terminal Vps28 were synthesized by Tsingke (Beijing, China). All DNA constructs were verified by sequencing.

### RNA interference

For RNA interference (RNAi), siRNAs targeting candidate genes and negative-control (NC) siRNA were synthesized by GenePharma (Shanghai, China). The sequences of the siRNAs for porcine Vps28 are as follows: siRNA 1, 5′-GGCUCAGAAAUCAGCUCUATT-3′; siRNA 2, 5′-GUCAGCUCCAUUGAUGAAUTT-3′, and siRNA 3, 5′-GAUGCUCUUUGACCUGGAATT-3′. The sequence of the NC siRNA is 5′-UUCUCCGAACGUGUCACGU-3′.

### Quantitative real-time PCR

RNAiso Plus (TaKaRa) was used to extract RNA from PK-15 cells, followed by reverse transcription to synthesize cDNA using 5× RT Master Mix (TaKaRa). Vps28, FMDV, beta-interferon (IFN-β), interleukin-6 (IL-6), and GAPDH transcript levels were quantified through quantitative real-time PCR (RT-qPCR). The primers specific for each gene were as follows: Vps28 (nucleotides 282–303), 5′-ATTCTGCCGCAAGTTCCGTCTG-3′ and 5′-CCTTGTCGTCCTTGATGGTGATGG-3′; FMDV 3D (nucleotides 30–49), 5′-CAAACCTGTGATGGCTTCGA-3′ and 5′-CCGGTACTCGTCAGGTCCA-3′; IFN-β (nucleotides 294–313), 5′-TGGCTGGAATGAAACCGTCA-3′ and 5′-AATGGTCATGTCTCCCCTGG-3′; IL-6 (nucleotides 374–395), 5′-ACCTGGACTACCTCCAGAAAGA-3′ and 5′-TTAGGGGTGGTGGCTTTGTC-3′; and porcine GAPDH (nucleotides 1,083–1,104), 5′-ACATGGCCTCCAAGGAGTAAGA-3′ and 5′-GATCGAGTTGGGGCTGTGACT-3′.

### Western blot

Cells were lysed to obtain the total protein fraction. Proteins were denatured with 1× SDS loading buffer, separated by SDS-PAGE, and transferred to NC membranes. Membranes were blocked for 1hour in 5% skim milk, incubated overnight with primary antibodies, washed with Tris-buffered saline–Tween (TBST) five times, and incubated with HRP-conjugated secondary antibodies for 1hour and again washed five times with TBST. Finally, the membranes were incubated with an enhanced chemiluminescence detection reagent (Thermo Fisher Scientific, Inc., Rockford, IL, USA) to visualize the protein band.

### Virus titration

Virus infectivity was titrated by endpoint dilution. Serially diluted samples were used to infect indicated cells in 96-well plates, and the TCID_50_ was calculated using the Reed–Muench method.

### Immunofluorescence assay

Cells infected with FMDV and/or transfected with specific plasmids were fixed with 4% paraformaldehyde for 30minutes at 37°C. After three washes with phosphate-buffered saline (PBS), the fixed cells were permeabilized with 0.1% Triton X-100 in PBS for 10minutes, then washed with PBS and blocked in 5% bovine serum albumin in PBS for 1hour. The cells were then incubated with an anti-FMDV porcine polyclonal antibody, an anti-HA rabbit monoclonal antibody, or an anti-Flag mouse monoclonal antibody overnight at 4°C, then washed with PBS three times and incubated with an FITC-conjugated rabbit anti-pig IgG secondary antibody, an FITC-conjugated rabbit anti-mouse IgG secondary antibody, or a TRITC-conjugated goat anti-rabbit IgG secondary antibody for 1hour at 37°C. The nuclei were stained with DAPI (Beyotime Biotechnolgy, Shanghai, China) for 10minutes and visualized. Images were captured with a laser scanning confocal microscope (Leica SP8; Leica, Solms, Germany).

### Transmission electron microscopy (TEM)

PK-15 cells mock-infected or infected with FMDV were fixed in a solution containing 3% glutaraldehyde and 2% paraformaldehyde in 0.1 M cacodylate buffer (pH 7.4) for 2hours at 4°C. Cells were washed and postfixed in 1% osmium tetroxide for 1hour and then incubated in 4% uranyl acetate for 2hours at room temperature. Samples were dehydrated at 4°C in alcohol. After treatment with alcohol–Epon (1:1) for 7hours at room temperature, samples were embedded in 100% Epon resin. Polymerization of the samples was performed in an oven at 35°C for 6hours, 45°C for 6hours, and 60°C for 24hours. The embedded samples were sliced into 80-nm (65-nm) sections and poststained with 4% uranyl acetate in H_2_O and lead citrate. Images were obtained using a Hitachi HT7800 transmission electron microscope.

### Immunoprecipitation assay

PK-15 cells were lysed with radioimmunoprecipitation assay lysis buffer (Beyotime Biotechnology) for 1hour on ice and then centrifuged at 15,000×*g* for 20minutes at 4°C. Supernatants were immunoprecipitated with the specific antibodies at 4°C overnight. The immune complexes were incubated with protein G-agarose beads (GE Healthcare, Chicago, IL, USA) for 2hours, washed six times with lysis buffer, and eluted in 1× SDS-PAGE sample buffer for western blot.

### Proteasome, lysosome, and caspase inhibitor assays

PK-15 cells were grown to a monolayer in six-well plates and infected with FMDV or mock for 1hour. Cells were then washed and incubated with DMEM supplemented with 1% FBS and the proteasome inhibitor MG132 (10 and 20 µM), the caspase inhibitor Z-VAD-FMK (10 and 50 µM), or the lysosomal inhibitor CQ (50 and 100 µM). After 11hours, cells were harvested for western blot analysis.

### Cell viability assay

PK-15 cells were seeded in 96-well plates and treated with the recombinant plasmids or siRNA duplexes for 24hours, or treated with MG132 (10 and 20 µM), the caspase inhibitor Z-VAD-FMK (10 and 50 µM), or the lysosomal inhibitor CQ (50 and 100 µM) for 24hours. The cytotoxic effects on PK-15 cells were evaluated via a Cell Counting Kit 8 (CCK8) assay. The reagent’s fluorescence was measured with a fluorescence microplate reader after incubation at 37°C.

### Statistical analysis

Data were obtained from at least three independent experiments for the quantitative analysis and were expressed as means ±SEs of the means. All statistical analyses were performed with a *t*-test or one-way analysis of variance. A *P*-value of <0.05 was considered a significant difference.

## SUPPLEMENTAL MATERIAL

Fig. S1 jvi.00181-23-s0001.pdf Cell viability evaluation of the recombinant plasmids, siRNA duplexes, and inhibitors.

ASM does not own the copyrights to Supplemental Material that may be linked to, or accessed through, an article. The authors have granted ASM a non-exclusive, world-wide license to publish the Supplemental Material files. Please contact the corresponding author directly for reuse.
