# Supplementary material for: Foot-and-mouth disease virus downregulates vacuolar protein sorting 28 to promote viral replication
Source: J Virol. 2023 Aug 11;97(8):e00181-23. doi: 10.1128/jvi.00181-23 (PMC10506468; doi:10.1128/jvi.00181-23)
Supplement: Fig. S1 — Cell viability evaluation of the recombinant plasmids, siRNA duplexes, and inhibitors. [file jvi.00181-23-s0001.pdf]

Supplemental materials

Fig. S1

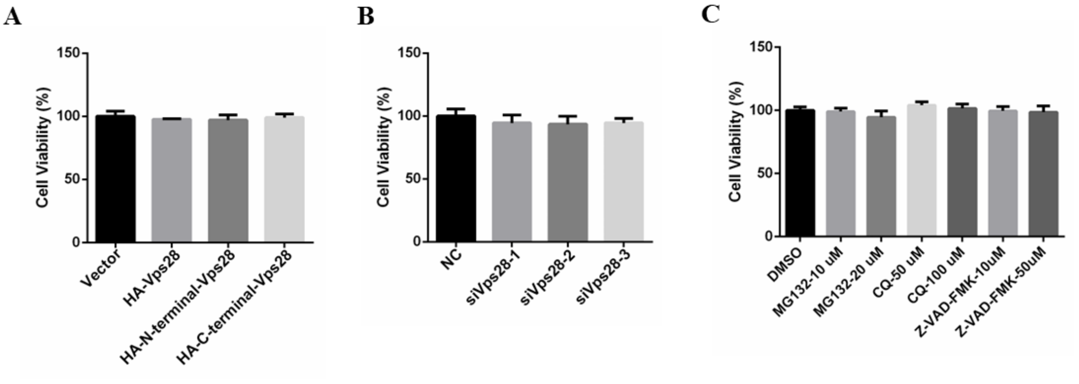

Fig. S1. Cell viability evaluation of the recombinant plasmids, siRNA duplexes, and inhibitors. (A to C) PK-15 cells were seeded in 96-well plates and treated with the recombinant plasmids (A) or siRNA duplexes (B) for 24h, or treated with MG-132 (10 and 20 µM), the caspase inhibitor Z-VAD-FMK (10 and 50 µM), or the lysosomal inhibitor CQ (50 and 100 µM) for 24h (C). The cytotoxic effects on PK-15 cells were evaluated via a CCK8 assay. The reagents fluorescence was measured with a fluorescence microplate reader after incubation at 37°C. No cytotoxicity was found in cells treated with the recombinant plasmids, siRNA duplexes, and inhibitors.
